# Development of patient specific, realistic, and reusable video assisted thoracoscopic surgery simulator using 3D printing and pediatric computed tomography images

**DOI:** 10.1038/s41598-021-85738-w

**Published:** 2021-03-18

**Authors:** Dayeong Hong, HaeKang Kim, Taehun Kim, Yong-Hee Kim, Namkug Kim

**Affiliations:** 1 Department of Biomedical Engineering, Asan Medical Institute of Convergence Science and Technology, Asan Medical Center, University of Ulsan College of Medicine, Seoul, Republic of Korea; 2 Department of Radiology and Convergence Medicine, Asan Medical Institute of Convergence Science and Technology, Asan Medical Center, University of Ulsan College of Medicine, 88 Olympic-ro 43 Gil, Songpa-gu, Seoul, 05505 Republic of Korea; 3 Department of Thoracic and Cardiovascular Surgery, Asan Medical Center, University of Ulsan College of Medicine, 88 Olympic-ro 43 Gil, Songpa-gu, Seoul, 05505 Republic of Korea

**Keywords:** Experimental models of disease, Paediatric research, Biomedical engineering

## Abstract

Herein, realistic and reusable phantoms for simulation of pediatric lung video-assisted thoracoscopic surgery (VATS) were proposed and evaluated. 3D-printed phantoms for VATS were designed based on chest computed tomography (CT) data of a pediatric patient with esophageal atresia and tracheoesophageal fistula. Models reflecting the patient-specific structure were fabricated based on the CT images. Appropriate reusable design, realistic mechanical properties with various material types, and 3D printers (fused deposition modeling (FDM) and PolyJet printers) were used to represent the realistic anatomical structures. As a result, the phantom printed by PolyJet reflected closer mechanical properties than those of the FDM phantom. Accuracies (mean difference ± 95 confidence interval) of phantoms by FDM and PolyJet were 0.53 ± 0.46 and 0.98 ± 0.55 mm, respectively. Phantoms were used by surgeons for VATS training, which is considered more reflective of the clinical situation than the conventional simulation phantom. In conclusion, the patient-specific, realistic, and reusable VATS phantom provides a better understanding the complex anatomical structure of a patient and could be used as an educational phantom for esophageal structure replacement in VATS.

## Introduction

Advances in techniques and instrumentation, such as video-assisted thoracoscopic surgery (VATS), in pediatric endoscopic surgery have enabled the performance of more complex and delicate procedures, even in small neonates. However, obtaining a comprehensive view of the operative field remains a challenge with existing techniques^1,2^. Clinically, one common application for thoracic surgery is the treatment of esophageal atresia with or without tracheoesophageal fistula (EATEF) in neonates^3^. Over the past 20 years, the number of minimally invasive surgical procedures in infants has significantly increased, including the repair of EATEF^4–7^. In 1995, the mean number of EATEF repairs performed by trainees in North America was 9.2^8^. By 2006, the mean number of repairs for trainees dropped to 4.4 in the United States^9^. With few opportunities for trainees to perform EATEF repair, VATS for EATEF repair may not be effectively taught to an advanced level of proficiency within a short training period. In this way, VATS for infants is tricky for experts as well as novices.

Recently, three-dimensional (3D) printing applications for reconstructing thoracic malformations in children have increased^10–13^, requiring patient-specific products to meet medical needs, one of personalized medical product that has been pioneered. The 3D printing process for phantom fabrication has multiple steps: (1) acquisition of high-quality computed tomography (CT) data on the anatomical structure to be modeled, (2) image processing to extract the region of interest from the anatomic structure, (3) 3D modeling from medical doctor needs, (4) quality assurance of the model to ensure its accuracy, (5) selection of the printing method and materials, and (6) printing of the phantom.

Several papers on patient-specific 3D printed phantoms have recently been published. For example, Hong et al. evaluated the accuracy of three different 3D printers by developing a personalized and realistic educational thyroid cancer phantom based on CT images: An evaluation of accuracy between three different 3D printers. That is produced a thyroid phantom for training residents on performing thyroid surgery^14^. Kyung et al. developed a 3D-printed kidney model for surgery planning, trainee teaching, simplifying the orientation of the target tissue, explaining risk structure, and predicting renal function post-operatively, thereby increasing the chances of improved surgical outcomes^15^.

With these advances in medical 3D printing, a suitable phantom for VATS for EATEF training must be created using the medical image as well as to aid in the planning of complex surgical procedures^16–21^. However, it was limited to create the thoracoscopic simulator described various tissue properties with disease model; For that reason, most simulators have lacked certain details and realism until now^22–24^. Therefore, our study aims are three folds including (1) to fabricate a patient-specific simulator with measurement between STL and simulators made by different 3D printers, (2) to fabricate realistic simulator with hardness measurement of various kinds of configurations, and (3) to fabricate a reusable thoracic simulator for VATS training in infant chest surgeries.

## Methods

This retrospective study was conducted in accordance with the principles of the Declaration of Helsinki and current scientific guidelines. The study protocol was approved by the Institutional Review Board of Asan Medical Center, South Korea. The requirement of informed consent from images was waived by the Institutional Review Board of Asan Medical Center (AMC). All methods were performed in accordance with the relevant guidelines and regulations.

**Figure 1:**
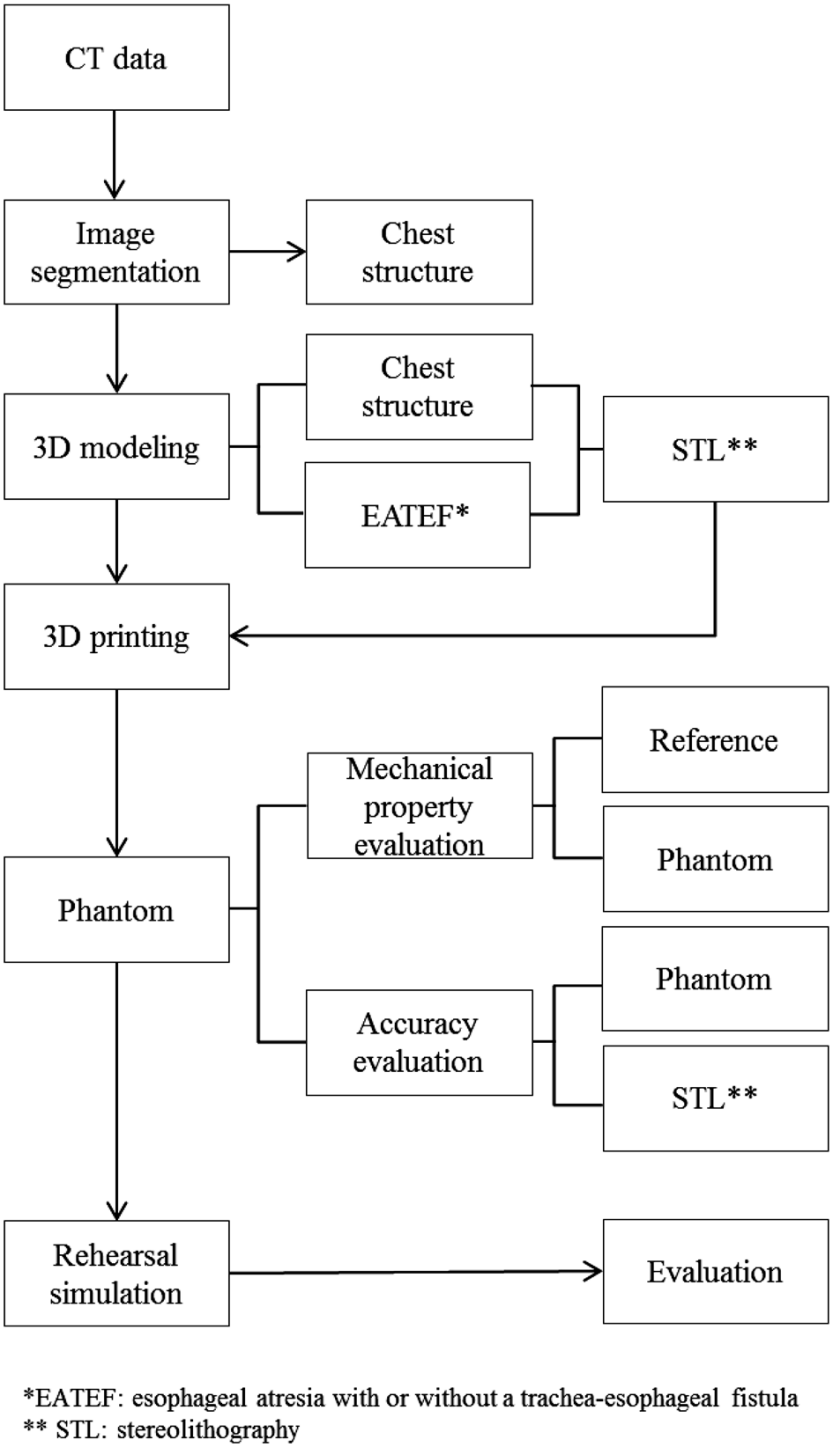
Flowchart of the procedure for making 3D-printed infant VATS phantom.

Medical imaging is required to produce patient-specific phantoms using 3D printing technology. Based on various medical images, such as CT and MRI, anatomical structures can be segmented and 3D modeled to create patient- or disease-specific 3D-printed models. Two types of 3D printers were used to fabricate actual phantoms with different materials. Shape accuracies and mechanical properties were evaluated to determine the final phantom, which was evaluated through simulation. The overall procedure is shown in Fig. 1.

### Data acquisition

Contrast-enhanced lung perfusion CT images from a 3-year-old patient were used as the source of the 3D model. The application details of the scanning protocol are as follows: a SOMATOM Definition Flash CT scanner (Siemens Medical Solutions, Forcheim, Germany) was used with a rotation time of 0.37 s, a pitch of 1 with a feed of 21 mm per rotation, and a tube voltage of 80 kV, 85 mA. The in-plane resolution of the CT data is 0.4 mm, and the distance between slices is 0.6 mm. An acceptable thin slice resolution (< 1 mm) is a key step because it has substantial effects on image resolution and noise, which in turn affect segmentation. The data included the entire lung tissue and chest wall with esophagus and trachea.

### 3D modeling for simulation

The 3D models of the lung, esophagus, trachea, and chest wall, including bone, muscle, and skin, were generated using the software Mimics and 3‐Matics (Materialise NV, Leuven, Belgium). Two-dimensional (2D) images were stacked by the software, yielding a threshold segmentation module with a set of tools, such as dilation, erosion, and boolean function, for the selection of pixels with gray values within a defined range of Hounsfield units representing the anatomical structures of interest. Subsequently, a region-growing algorithm was used to separate these structures of interest from the surrounding tissue. If the segmented anatomical structures were not clearly distinguished by a marked contrast in pixel gray values, then the desired pixels were manually drawn on each 2D CT image. 3D volume rendering models could also be refined by a volume-sculpting operation to achieve accurate representation of the desired organs in thoracic structures. Finally, the segmented 3D images were converted into stereolithography (STL) format consisting of a triangular surface mesh structure by the software. The 2 mm wall thickness of the airway and esophagus was modeled by an outside offset function, and the fistula that was invisible in CT images was created using Meshmixer (Autodesk, Inc., Toronto, Canada). After that, seven artificial holes under each rib (third to eighth) were created for the placement of VATS ports. The scale of the model was reduced to 80% to match that of 9- to 12-month-old infants according to the Korean standard size. The diameter of VATS port holes was set to 12 mm by Magics RP (Materialise Inc., Leuven, Belgium). Finally, the unnecessary left half of the chest wall was cut out to reduce printing time and cost as well as for easy fixing onto the base panel^21^.

**Figure 2:**
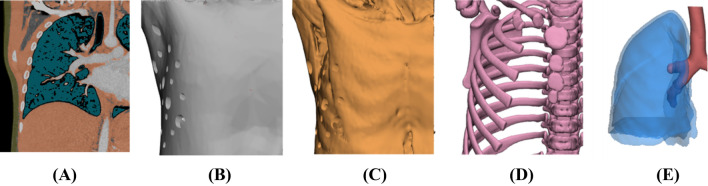
CT image segmentation and 3D modeling for 3D rehearsal simulation phantom: (**A**) left lobe of the lung, (**B**) skin, (**C**) muscle, (**D**) bone, and (**E**) airway and lung. (Mimics 20.0 and 3‐matic 12.0, Materialise NV, Leuven, Belgium).

To ensure the durability and efficiency of the simulator, it was divided into fixed and replaceable parts. The fixed part is the chest wall including the skin, muscle, bone structures, and esophagus model; the lung was separated as a replaceable part. The simulator was effectively applied to practice the various anatomical differences depending on the disease type (Fig. 2).

### 3D printing

The pediatric phantoms were produced using two types of 3D printers. For realistic simulation, holes of various sizes in the phantoms were implemented in the skin and muscle structure. Various types of airways with tracheoesophageal fistula were also designed and printed on reusable purpose.

#### Fused deposition modeling (FDM) 3D printer

The Good boT 6300MP printer (3D KOREA, CO, Korea) was used, which printed on KFLX28 filament. Conventional FDM 3D printers have difficultly printing on multicolored materials; however, the advantage of this printer is that it can print out various colors simultaneously. In particular, the KFLX28 filament used in simulator production had various colors and high flexibility and elasticity compared with existing acrylonitrile butadiene styrene (ABS) and polylactic acid (PLA). Using this feature, the lung, skin, muscle, bone, airway, and esophagus were printed on a replaceable structure. The skin and airways were designed specifically for the disease and patient and can be replaced.

#### PolyJet 3D printer

Using the Objet500 Connex3 printer (Stratasys, CO, USA), Vero color (hard material), and Aglius (soft material) can be effectively combined and printed immediately. In fact, the combination of PolyJet printing materials enabled the control of the elongation and hardness and reflected various elongation and hardness compared with FDM. Moreover, unlike FDM, colors and materials can be combined to generate structures of various hardness immediately. Therefore, the bones, muscles, and skin can be produced all at once, as well as produced separately to replace the lungs and airways.

### Material properties

Using the refined 3D models and various materials, the thoracoscopic surgery simulator was produced using Good boT 6300MP and Objet 500 Connex 3. The two printers are often used for medical printing because the different colors of anatomical structures can be informative to clinical personnel.

The FDM 3D printer is highly commercialized and uses various types of materials, which are inexpensive compared with those of other types of printers. However, the hardness of these materials is difficult to control, and the surface is not smooth because the supporter is needed to overcome the inertia. PolyJet printers require more expensive materials and equipment compared with other printers, but the accuracy is high. In addition, the hardness can be adjusted by combining various types of materials with different elongations and hardness. This 3D printer offers the ability to print with dual materials to provide a wide range of soft, rubber-like models using Shore A hardness, elongation at break, tear resistance and tensile strength, and rigid colored PolyJet photopolymers with multi materials from Vero color (hard material) and Aglius (soft material) (Table 1).

**Table 1:** Comparison of two types of 3D printing: FDM and PolyJet.

	FDM	PolyJet
Cost	Low cost	High cost
Materials	Thermoplastic	Photopolymer
Feature	Multi-color, need to support	Multi-color, multi-material, soft, high accuracy

*FDM* fused deposition modeling.

To implement the hardness of the anatomical structure, the elongation of each material was referenced (KFLX28 330 to 560%, Agilus and Vero 220 to 270%), and the hardness was directly measured. The KFLX28 material of the FDM fitting printer was measured for hardness by thickness, and the PolyJet type was measured by the material mixing ratio between Agilus and Vero.

### Accuracy evaluation

**Figure 3:**
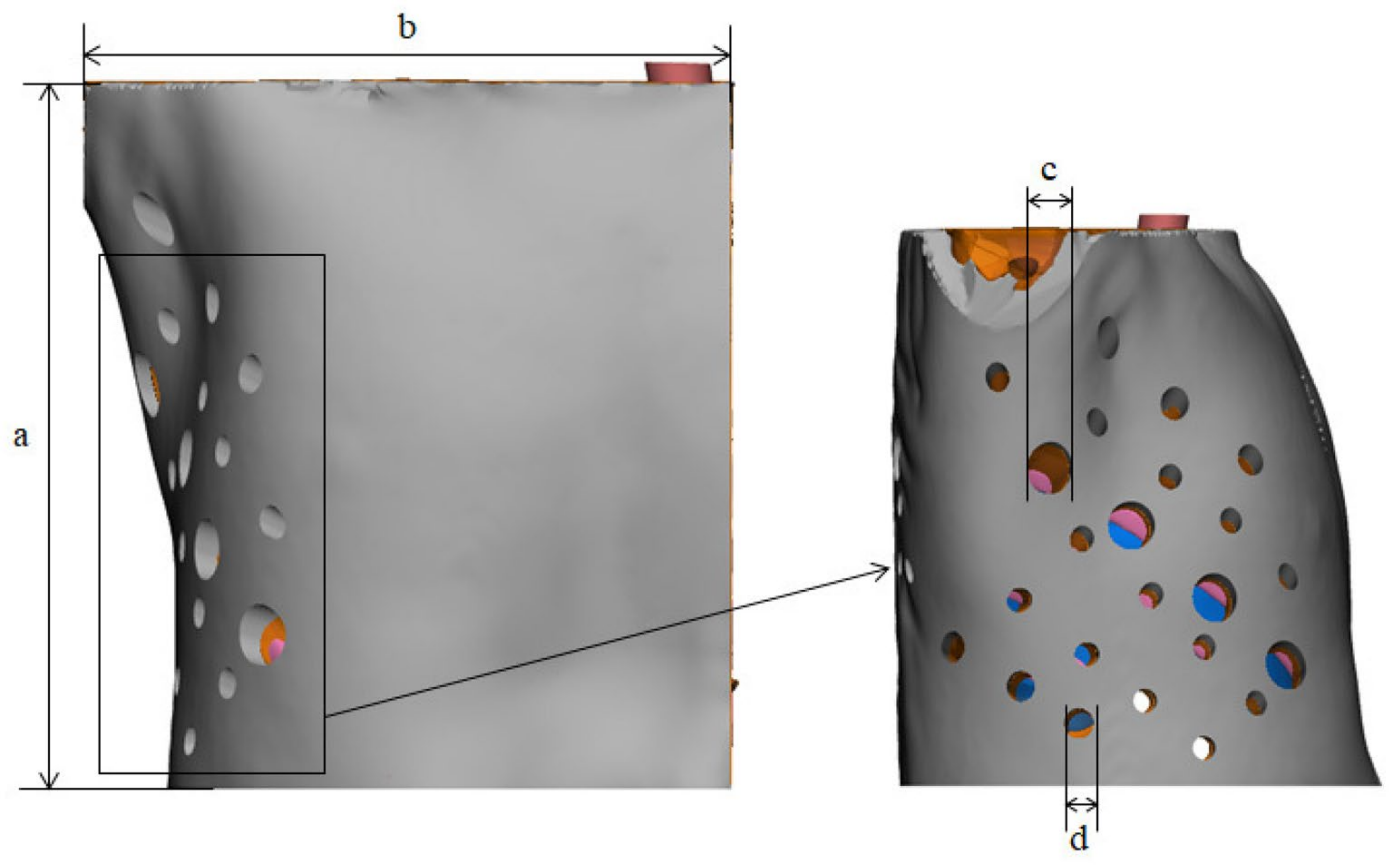
STL file with four landmarks specified for evaluating measurement error: (**a**) height, (**b**) width, (**c**) diameter of the VATS hole, and (**d**) diameter of another VATS hole. (3 matic 12.0, Materialise NV, Leuven, Belgium).

The phantom was printed by the two types of 3D printers from the same STL file. To compare the accuracy of each 3D-printed phantom with that of the STL modeling file, we assigned the same landmarks to four locations, which were measured by two researchers using Vernier calipers (Fig. 3), for a total of eight measurements. Bland–Altman analysis was conducted to evaluate the STL file and 3D-printed phantoms using XLSTAT 2020 software. Paired t-test was used to compare the differences between the STL file and two types of phantoms using the SPSS software (trial version 25.00; IBM).

### Simulation for VATS

A simulation was conducted for the VATS procedure of one infant with EATEF with this simulator and with conventional vendor provided simulator (Medtronics, MN, USA). One expert thoracic surgeon with over 15 years of experience evaluated the effectiveness of the 3D printed phantom in VATS for infant thoracic surgery. For the clinicians, the effectiveness of this simulator to conventional one was evaluated: (a) prediction of the effectiveness before using our phantom, (b) effectiveness after using our phantom comparing to the conventional one, and (c) the reality of the EATEF simulator was used.

## Results

The 3D modeling process performed by an experienced operator consumed approximately 5–7 h (CT image to STL). Modeling and designing were the most challenging steps (3–4 h) owing to various issues, such as resolution, artifacts of CT images, and small anatomical structures (i.e., small size of the infant anatomy) in the CT images. The export of the reconstructed mesh surface to the STL file was simple and consumed only a few minutes. However, triangular mesh simplification with Meshlab 2020 (Visual Computing Lab, ISTI–CNR, Italy) and nonmanifold surface error fixing with Meshmixer consumed approximately 20 min.

**Figure 4:**
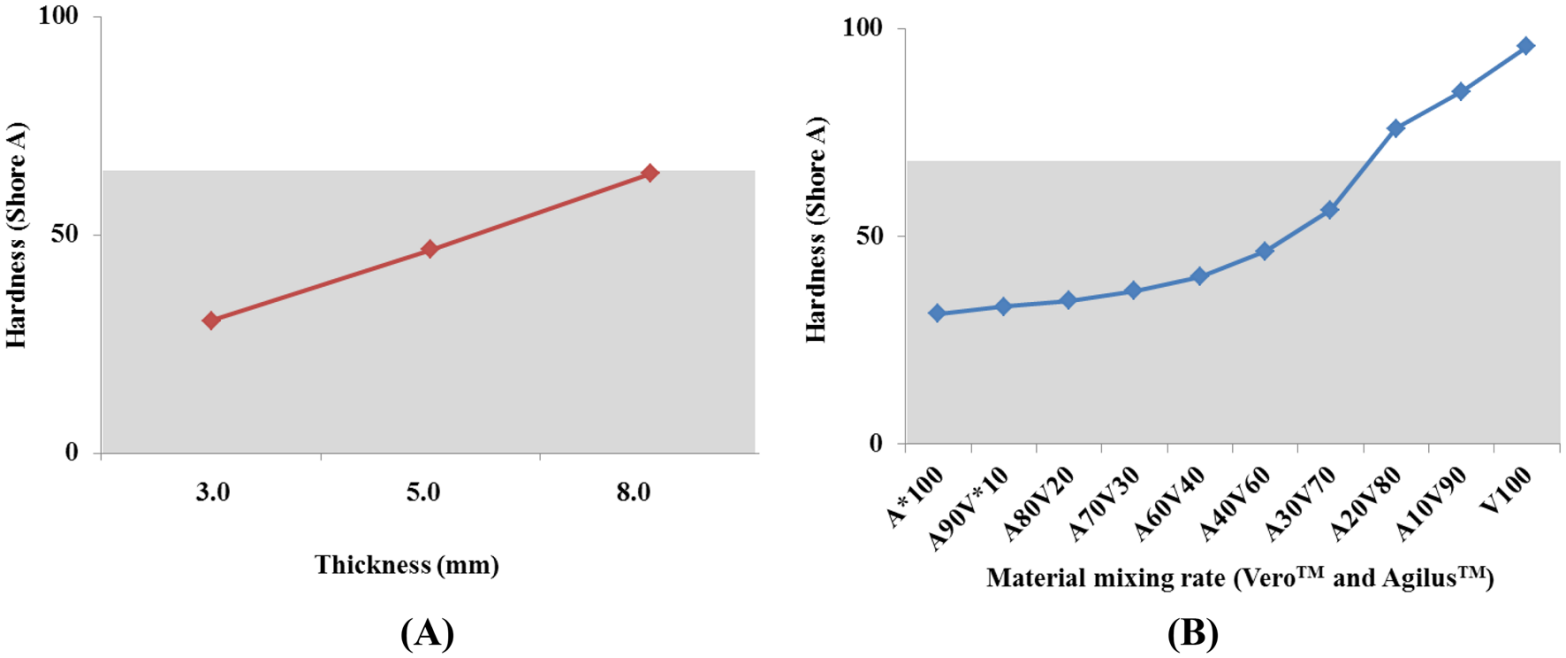
Comparison of mechanical properties of 3D printing materials. The gray zone in both graphs is human skin to shore A hardness (mean) ranging from 0 to 65. (**A**) Hardness with different thickness values of FDM printing materials. (**B**) Hardness according to the mixing ratio of two PolyJet materials. (A*: Agilus, V*: Vero).

Prior to implementation of the simulation phantom, the hardness of the materials used in the two printing methods was evaluated. The result shows that the materials could not be mixed in the FDM printing process and the hardness of materials differed according to the thickness of the printed object. At this time, the greater the thickness, the higher the hardness. In the case of PolyJet printing, the materials could be mixed. Therefore, the material mixture was printed and measured in 10 steps. The higher the proportion of Vero material (or the lower the proportion of Agilus material), the higher the hardness (Fig. 4).

**Figure 5:**
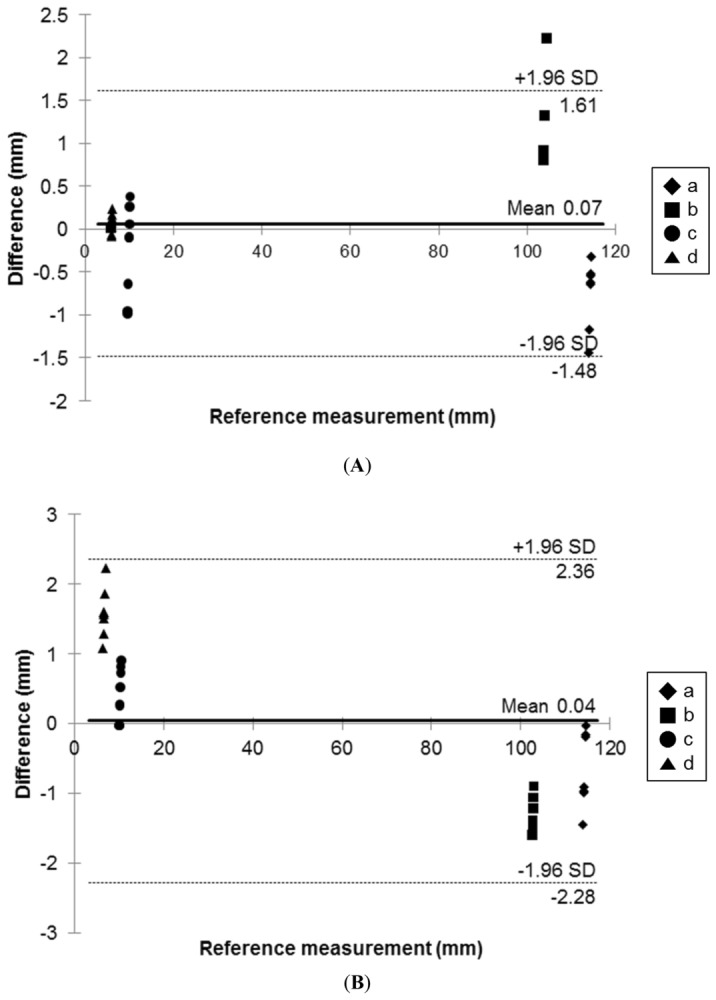
Bland–Altman analysis used to evaluate differences between the STL file (standard) and the two 3D-printed phantoms. (**A**) STL vs FDM, (**B**) STL vs PolyJet. The chosen landmarks were (**a**) height, (**b**) width, (**c**) diameter of VATS hole, and (**d**) diameter of another VATS hole in Fig. 4.

The corresponding metrics in the original STL file were compared with the physical measurements and evaluated using a Bland–Altman plot (Fig. 5 and Table 2). In 3D printing with FDM, the mean ± standard deviation (SD) of the differences was 0.53 ± 0.46 mm (limits of agreement from − 1.5 to 1.6 mm) (Fig. 5A). In 3D printing with PolyJet, the mean ± SD was 0.98 ± 0.55 mm (limits of agreement from − 2.3 to 2.4 mm) (Fig. 5B). All of the measurements were within the limits of agreement.

**Table 2:** Comparison of FDM and PolyJet phantoms.

	FDM	PolyJet
Modality	Good boT 6300MP (3D KOREA, CO, Korea)	Objet500 Connex3 (Stratasys Ltd.)
Materials	KFLX28 filament	Vero, Agilus
Printing cost	$500	$1600
Printing time	72 h	16 h

*FDM* fused deposition modeling.

**Figure 6:**
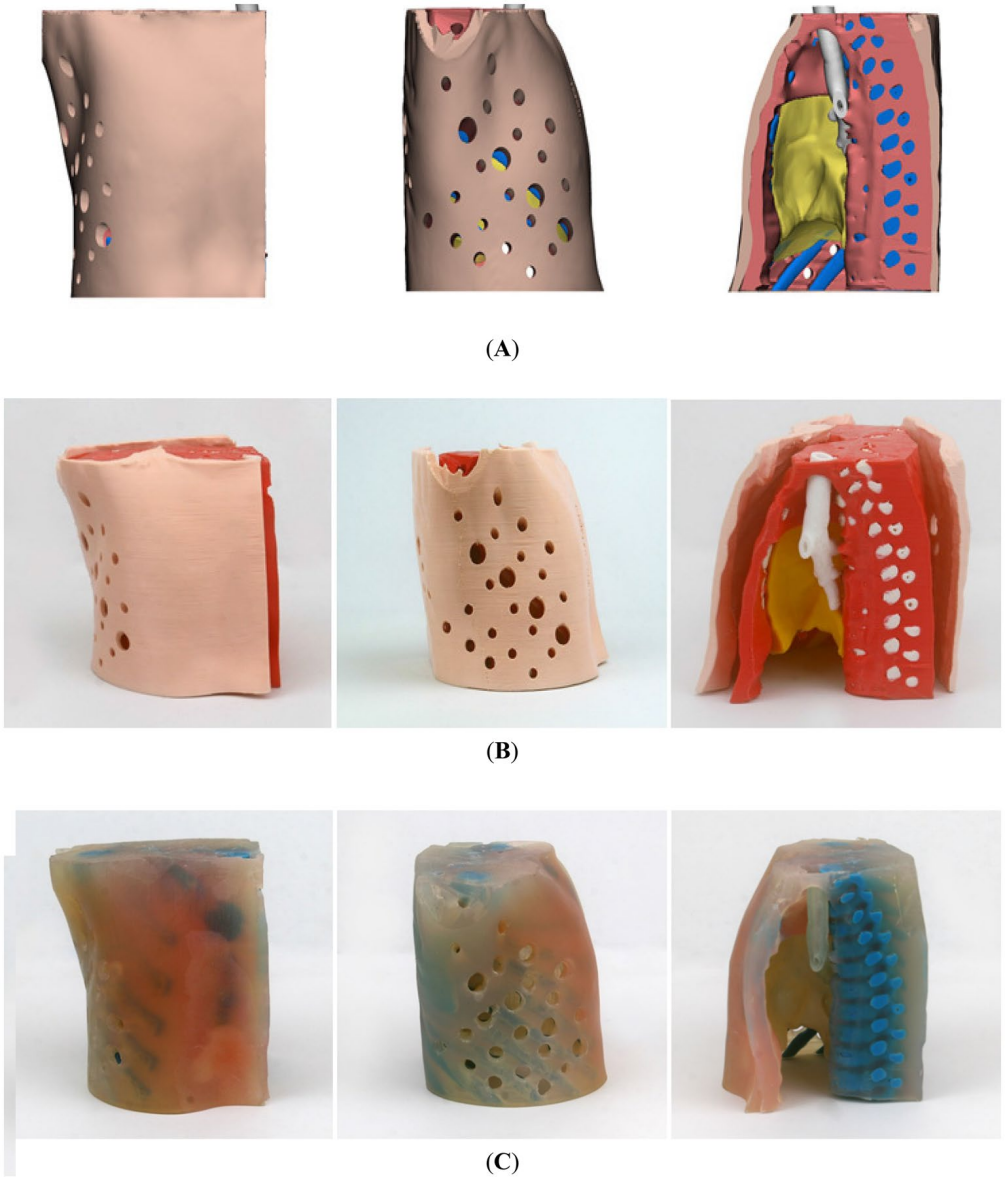
Pediatric thoracic phantom from 3D modeling and made with two types of 3D printers: (**A**) 3D modeling, (**B**) FDM, and (**C**) PolyJet. (3‐matic 12.0, Materialise NV, Leuven, Belgium).

Figure 6 shows that the infant thoracic phantoms fabricated by two types of 3D printing. With FDM, the anatomical structure was fabricated by assembly, and the hardness of the material was adjusted based on the thickness. In addition, unlike PolyJet printing, since there is no transparent material, different colors were used to distinguish the anatomical structure. The phantom printed by the FDM printer consumed approximately 72 h, while the postprocessing consumed approximately 96 h. The 3D printing cost was $800. The PolyJet printer can control the hardness using material mixing. Therefore, various diseases can be reflected by replacing the airways and lungs. By mixing the materials, similar physical properties can be realized by reflecting various hardness values. The phantom printed by the PolyJet printer consumed approximately 16 h at a cost of approximately $1,600 (Table 3).

**Table 3:** Comparison of the reference measurements between the two types of 3D-printed phantoms.

Reference measurement—3D-printed phantom	3D printing method	
	FDM	PolyJet
Mean absolute difference (mm)	0.53 ± 0.50	0.98 ± 0.55
Mean relative difference (%)	1.34 ± 0.85	8.15 ± 12.33

*FDM* fused deposition modeling.

**Figure 7:**
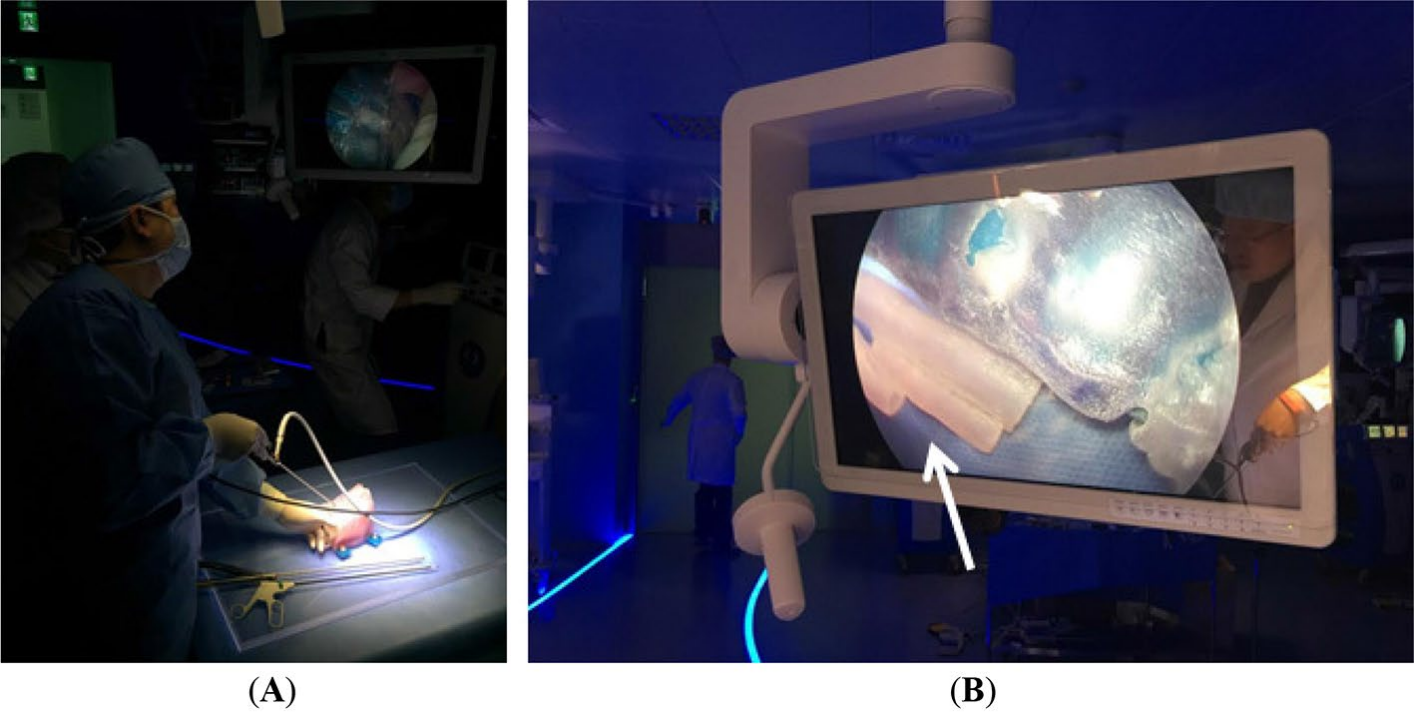
VATS simulation with esophageal atresia with a tracheoesophageal fistula using 3D-printed phantom: (**A**) a surgeon’s simulation of the 3D-printed phantom and (**B**) viewing the video screen (thick white arrow to indicate esophageal atresia with a tracheoesophageal fistula).

The simulator for VATS training was evaluated by an expert thoracic surgeon (Fig. 7). The informed consent was obtained for identifying images 7(A) and 7(B) to publish the images. Based on the questionnaire, the surgeon responded as (a) prediction of the effectiveness before using our phantom, 3; (b) effectiveness after using our phantom comparing to the conventional one, 5; and (c) the reality of the EATEF simulator, 5. In addition, the surgeon commented that it is easy to plan the pre-operation and the time for surgery is reduced, and both types of phantoms were qualitatively rated as very useful in training and reflecting VATS than non-simulated before surgery. Especially, the texture of the anatomical structure could be realistically reflected by using the polyjet printing technology that can realize various hardness.

## Discussion

Most of the currently commercialized endoscopic surgical simulators are designed for abdominal surgery, and there are few types for thoracic surgery. Recently, with the introduction of VR, research for commercialization is in progress, but there is no VATS simulator for pediatric surgery except a general thoracic simulator. In particular, esophagus and trachea are small and special organs, and related simulators do not exist. In the case of pediatric, unlike adults, it is difficult to obtain an appropriate experience because the subject of minimally invasive surgery such as VATS is very rare due to their small size. Therefore, it could be meaningful that thoracic surgeons and pediatric surgeons can use the simulator to provide sufficient experience for minimally invasive surgery in pediatric.

Moreover, because patients with EATEF are rare, determining whether efficient surgery is possible with VATS is not easy. The purpose of this study is to create a realistic simulator for a patient using 3D printing technology for VATS training in infant chest surgery. Therefore, to create a patient-specific phantom, we compared two 3D printing technologies and tried to devise a more realistic phantom. However, all the hardness suitable for human skin or tissue is difficult to determine. The properties of human skin and muscle are too variable to simulate and standardize. Several studies have shown that the mechanical properties of human skin depend on a variety of factors, such as age, gender, and location of the site^25–27^. Therefore, this study was conducted with reference to known mechanical properties of human anatomical features. The results of Blend–Altman analysis showed that the accuracies of both the phantoms and STL modeling files were acceptable for EATEF simulation in VATS. In general, it is known that PolyJet printing is much more accurate and expensive. However, in this study, some measurements using PolyJet have lower accuracy. In particular, the measured value of b (diameter of width) in Fig. 5 is lower than that of the FDM phantom. Because the ratio of the Agilus material used for the skin and muscle was high and the soft material such as Agilus of the PolyJet could not guarantee the shape accuracy^28^, an error likely occurs in measurements using Vernier calipers. For the same reason, the measurement range of PolyJet is wider than that of FDM in d (diameter of vats hole) in Fig. 5, which was made by combining Agilus with low-hardness PolyJet materials^28^.

As several holes were marked on the skin to allow the port to provide various viewpoints of VATS endoscopies and anatomical variations of EATEF were replaceable, the phantom could be reused for various purposes in VATS. Even in cases requiring tumor or lung resection, such as tracheoesophageal fistula, mediastinal tumor, or lung tumor, if the internal model of the disease is generated, the thorax model itself can be recreated and simulated. However, for a more accurate procedure, a method of manufacturing a patient- and disease-specific simulator based on the medical image of the patient is available, which could be expensive. This phantom can also be used for various educational purposes, such as educating inexperienced junior surgeons or patients scheduled for surgery to provide them a better understanding of the disease and surgical procedures. The simulator has the potential to aid in pre-operative planning and serve as a surgical guide. The phantom for educational purposes can also be applied to augmented reality and virtual reality as well as in simulation. Ten general thoracic surgeons used the simulator in this study. They (major, full-time, young professor) experienced VATS lung surgery on pigs in an animal lab as a beginner course. Then, as an advanced course, the VATS simulator for children of this study was used, and their feedback on this was delivered through discussion in the field. As a result, the difficulty of the procedure was very high, but everyone agreed that it was worth using it for rehearsal training in pediatric VATS surgery. Also, the evaluation of EATEF simulation by a surgeon with over 15 years of experience was positive, and the simulation of the 3D-printed phantom has a number of advantages over conventional surgery. By determining the complex anatomical variations around EATEF in advance, difficult surgeries can be planned through rehearsal simulation, thus reducing the surgeons’ effort, time, and burden. Moreover, this simulation can be extended to adult patients and for other procedures, such as VATS lobectomy. This type of preoperative experience will enable surgeons to perform thoracoscopic surgery efficiently and safely and improve surgical outcomes by allowing them to recognize the critical surrounding structures.

This study has several limitations. First, simulation was performed by only one surgeon who reviewed the materials and clinical acceptability of the phantom. Since our paper focused on development of a simulator, this procedure was very difficult and the number of operations was not large, it was not possible to gather opinions from various surgeons. Therefore, we cannot assume that these findings will be reproduced by all surgeons. More surgical cases and more surgeons' opinions will be collected in further studies. In addition, given that this study was based on the CT image of only one pediatric patient, there is a limit to reflecting the diversity of the anatomical abnormalities of EATEF, and it can be applied only in the training of thoracic surgeons specializing in pediatrics. In the future, phantoms of many patients should be fabricated and evaluated by multiple surgeons. If simulation is required for surgeons of various experiences, various conclusions can be drawn. By supplementing the size or internal anatomy, the simulation phantom training could be By supplementing the size or internal anatomy, the simulation phantom training could be extended to various kinds of thoracoscopic phantoms that reflect the age, sex, and specific diseases of both children and adults. At present, available 3D printing materials are not completely satisfactory and are different from the human thoracic portion in terms of their mechanical properties, including hardness, and elasticity. Thus, to produce more realistic simulators, research must be conducted by developing and combining silicone casting and various 3D printing materials.

## References

[1] McKenna RJ, Houck W, Fuller CB (2006). Video-assisted thoracic surgery lobectomy: experience with 1,100 cases. Ann. Thorac. Surg..

[2] Jensen K (2017). Using virtual reality simulation to assess competence in video-assisted thoracoscopic surgery (VATS) lobectomy. Surg. Endosc..

[3] Wei S, Saran N, Emil S (2017). Musculoskeletal deformities following neonatal thoracotomy: long-term follow-up of an esophageal atresia cohort. J. Pediatr. Surg..

[4] Burjonrappa S, Youssef S, St-Vil D (2011). What is the incidence of Barrett's and gastric metaplasia in esophageal atresia/tracheoesophageal fistula (EA/TEF) patients?. Eur. J. Pediatr. Surg..

[5] Gonzalez D, Paradela M, Garcia J, dela Torre M (2011). Single-port video-assisted thoracoscopic lobectomy. Interact. Cardiovasc. Thorac. Surg..

[6] Holder TM, Cloud DT, Lewis JE, Pilling GP (1964). Esophageal atresia and tracheoesophageal fistula: a survey of its members by the Surgical Section of the American Academy of Pediatrics. Pediatrics.

[7] Little DC (2003). Long-term analysis of children with esophageal atresia and tracheoesophageal fistula. J. Pediatr. Surg..

[8] Rowe MI, Courcoulas A, Reblock K (1997). An analysis of the operative experience of North American pediatric surgical training programs and residents. J. Pediatr. Surg..

[9] Sømme S, Bronsert M, Kempe A, Morrato E, Ziegler M (2012). Alignment of training curriculum and surgical practice: implications for competency, manpower, and practice modeling. Eur. J. Pediatr. Surg..

[10] Burke RP, Wernovsky G, van der Velde M, Hansen D, Castaneda AR (1995). Video-assisted thoracoscopic surgery for congenital heart disease. J. Thorac. Cardiovasc. Surg..

[11] de Lagausie P (2005). Video-assisted thoracoscopic surgery for pulmonary sequestration in children. Ann. Thorac. Surg..

[12] Cano I, Antón-Pacheco JL, García A, Rothenberg S (2006). Video-assisted thoracoscopic lobectomy in infants. Eur. J. Cardiothorac. Surg..

[13] Koontz CS, Oliva V, Gow KW, Wulkan ML (2005). Video-assisted thoracoscopic surgical excision of cystic lung disease in children. J. Pediatr. Surg..

[14] Hong D (2019). Development of a personalized and realistic educational thyroid cancer phantom based on CT images: an evaluation of accuracy between three different 3D printers. Comput. Biol. Med..

[15] Kyung YS, Kim N, Jeong IG, Hong JH, Kim C-S (2019). Application of 3-D printed kidney model in partial nephrectomy for predicting surgical outcomes: a feasibility study. Clin. Genitourin. Cancer.

[16] Mowry SE, Jammal H, Myer C, Solares CA, Weinberger P (2015). A novel temporal bone simulation model using 3D printing techniques. Otol. Neurotol..

[17] Chae MP (2015). Emerging applications of bedside 3D printing in plastic surgery. Front. Surg..

[18] Weinstock P, Prabhu SP, Flynn K, Orbach DB, Smith E (2015). Optimizing cerebrovascular surgical and endovascular procedures in children via personalized 3D printing. J. Neurosurg. Pediatr..

[19] Barber SR (2016). 3D-printed pediatric endoscopic ear surgery simulator for surgical training. Int. J. Pediatr. Otorhinolaryngol..

[20] Tai BL (2015). Development of a 3D-printed external ventricular drain placement simulator. J. Neurosurg..

[21] Parthasarathy J (2014). 3D modeling, custom implants and its future perspectives in craniofacial surgery. Ann. Maxillofac. Surg..

[22] Jensen K, Ringsted C, Hansen HJ, Petersen RH, Konge L (2014). Simulation-based training for thoracoscopic lobectomy: a randomized controlled trial. Surg. Endosc..

[23] Meyerson SL, LoCascio F, Balderson SS, D'Amico TA (2010). An inexpensive, reproducible tissue simulator for teaching thoracoscopic lobectomy. Ann. Thorac. Surg..

[24] Barsness KA, Rooney DM, Davis LM (2013). Collaboration in simulation: the development and initial validation of a novel thoracoscopic neonatal simulator. J. Pediatr. Surg..

[25] Falland-Cheung L, Pittar N, Tong D, Waddell JN (2015). Investigation of dental materials as skin simulants for forensic skin/skull/brain model impact testing. Forensic Sci. Med. Pathol..

[26] Merkel PA (2008). Validity, reliability, and feasibility of durometer measurements of scleroderma skin disease in a multicenter treatment trial. Arthritis Care Res..

[27] Jussila J, Leppäniemi A, Paronen M, Kulomäki E (2005). Ballistic skin simulant. Forensic Sci. Int..

[28] Kim T (2020). Accuracies of 3D printers with hard and soft materials. Rapid Prototyping J..

